# Defining the Field of Behavioral Medicine: A Collaborative Endeavor

**DOI:** 10.1007/s12529-016-9616-1

**Published:** 2016-11-23

**Authors:** Joost Dekker, Adrienne Stauder, Frank J. Penedo

**Affiliations:** 1 Department of Psychiatry and Department of Rehabilitation Medicine, VU University Medical Center, PO Box 7057, Amsterdam, 1007 MB The Netherlands; 2 Jining Medical University, Jining, China; 3 Faculty of Sports and Health Sciences, University of Jyväskylä, Jyväskylä, Finland; 4 Institute of Behavioral Sciences, Semmelweis University Budapest, Budapest, Hungary; 5 Departments of Medical Social Sciences, Psychology and Psychiatry and Behavioral Sciences, Northwestern University, Chicago, IL USA

**Keywords:** Behavioral medicine, Definition, Scope

## Abstract

**Purpose:**

To respond to comments on our proposal for an update of the definition and scope of behavioral medicine.

**Methods:**

We identify common themes in the comments and provide a response.

**Results:**

We discuss the relationship of behavioral medicine to other disciplines and fields, the scope of behavioral medicine, and issues related to the application of behavioral medicine.

**Conclusion:**

Based on the comments of our esteemed colleagues and our reflection on those comments, we now offer the following refined definition and scope of behavioral medicine. *‘Behavioral medicine can be defined as the field characterized by the collaboration among multiple disciplines concerned with the development and integration of biomedical and behavioral knowledge relevant to health and disease, and the application of this knowledge to prevention, health promotion, diagnosis, treatment, rehabilitation, and care. The scope of behavioral medicine extends from bio-behavioral mechanisms (i.e. the interaction among biomedical, psychological, social, societal, cultural and environmental processes related to health and disease), to clinical diagnosis and intervention, and to public health’*. We propose to use this refined definition and scope as the starting point for seeking further input from the ISBM member societies.

We are very grateful for the thoughtful and constructive comments from our colleagues on our recent proposal for an update of the definition and scope of behavioral medicine [1–6]. These comments underscore the need for an update. These comments also show that additional perspectives need to be taken into account - perspectives of the colleagues who provided comments, and perspectives of others not yet involved in the discussion. Below, we reply to the comments, resulting in a more conceptually sound and refined proposal. We offer this refined proposal as a starting point for further discussions.

## The Relationship to Other Disciplines and Fields

We agree that the definition of behavioral medicine “needs to be inclusive instead of exclusive” [4], that we should not exclude related disciplines [6] but rather “embrace” scientists working in related fields [5], that there are “no clear lines between disciplines” [3], and that “synergy” among disciplines is a defining and important feature of the field of behavioral medicine [2]. It was not our intention to define behavioral medicine as a discipline separate from other disciplines, nor to exclude specific disciplines from contributing to the field. We agree that behavioral medicine is indeed a *scientific field*, which derives its vitality and strength from *strong collaboration among multiple disciplines* [2, 3].

On the other hand, there is an urgent need to clearly define the field of behavioral medicine  - to state what it is and what it is not. We must be able to justify the field of behavioral medicine as offering unique and important approaches to the study of issues pertaining to health and disease; therefore, we agree with Weiss’ suggestion that we should “not conclude that everything we do is ‘behavioral medicine’” [2]. The term and the field of behavioral medicine would become meaningless if we would include “everything”, and behavioral medicine would lose its current vitality and strength. Exactly with the intention of increasing its appeal to many disciplines, we need to clearly define and operationalize the field of behavioral medicine, and describe its unique features and contributions.

In an attempt to be inclusive while also setting boundaries, we propose to describe behavioral medicine ‘as the field characterized by the collaboration among multiple disciplines concerned with the development and integration of biomedical and behavioral knowledge relevant to health and disease’. This definition emphasizes collaboration among biomedical and behavioral disciplines. Collaboration is the defining characteristic of behavioral medicine, while both “biomedical” and “behavioral” are sufficiently broad categories to cover contributions from a wide range of specific disciplines. It should be noted that we gratefully adopted “multiple disciplines” [3], avoiding terms such as “multidisciplinary,” “interdisciplinary,” and “transdisciplinary” which refer to a continuum of increasing involvement of multiple disciplines [7]. The approach of ‘behavioral medicine as the field characterized by the collaboration among multiple disciplines’ emphasizes collaboration, leaving it open how exactly the various disciplines work together.

We believe that this definition makes it possible to distinguish behavioral medicine from related fields such as mental health, health psychology, and psychosomatic medicine. Further work on how to exactly describe the distinction between behavioral medicine and related fields seems to be indicated. We believe that our previous proposal [1] and the comments from our colleagues provide valuable input for that endeavor.

While we appreciate the thoughtful comments regarding a potential dualistic flaw in our definition, we do not believe that making a distinction between behavioral medicine and mental health is “an inadvertent recreation of Cartesian dualism” [2], or that making this distinction leads to the exclusion of mental disorders as target conditions in behavioral medicine [6]. We emphasize the integration of biomedical and behavioral knowledge as a defining characteristic of behavioral medicine; and in agreement with Nater [5], we regard behavioral medicine as primarily focusing on the role of behavior in somatic disease (with a broad definition of “behavior”, which includes mental disorders and processes; see below), and mental health primarily as focusing on mental disorders. Neither behavioral medicine nor mental health can ignore the strong link between mind and body. However, we suggest that merely targeting mental health issues among individuals with a chronic disease, for example, with no attention to how social, behavioral, biological, and other determinants of health and disease intersect and contribute to physical health outcomes is not behavioral medicine. We acknowledge that terms such as “somatic”, “physical” and “mental” seem to suggest a dualism; unfortunately, we as well as the entire field seem to lack better terms.

Lau [4] called attention to the fact that a number of the ISBM member societies used behavioral health in naming their society, instead of behavioral medicine. We agree that this is an important issue. Indeed, the definition of behavioral medicine refers to health: ‘the development and integration of biomedical and behavioral knowledge relevant to *health* and disease’. Whether there is a need to mention “health” in the name of our field and our society is an issue for further discussion.

### The Scope of Behavioral Medicine

We proposed the scope of behavioral medicine to extend “from bio-behavioral mechanisms (i.e. the interaction of biomedical processes with psychological, social, societal, cultural and environmental processes), to clinical diagnosis and intervention, and to public health” [1]. We proposed to clarify and broaden the meaning of “bio-behavioral mechanisms” from exclusively biomedical mechanisms (e.g., stressors and stress responses) to a broad range of processes in the psychological, social, societal, cultural, and environmental domain, that contribute to health and disease; this may or may not include attention to specific biological and disease-related systems.

This proposal leads to various responses. On the one hand, Weiss [2] reminds us that the original emphasis on biomedical mechanisms was intentional; it was and probably still could be an attempt to attract the skeptical biomedical disciplines. On the other hand, Lau [4] proposes to broaden the scope even further: “it is not necessary to involve biomedical processes in behavioral medicine”. He refers to research on how policy and physical environment interact with cognitions to determine levels of physical activity, as an example. Lau [4] proposes to describe the scope as follows: “… from bio-psychosocial-behavioral mechanisms (i.e. the interactions *among* biomedical processes, behavioral processes, psycho-social processes, and environmental processes related to health)”.

Taking into consideration how the field of behavioral medicine is evolving (as witnessed at the International Congress of Behavioral Medicine, and in papers in the International Journal of Behavioral Medicine, for example), we agree that an exclusive focus on biomedical mechanisms is no longer appropriate. Psychological and social mechanisms are strongly related to health and disease, and these mechanisms belong to the core emphasis of behavioral medicine. On the other hand, we are wary to drift away from the strong link to health and disease: The integration of biomedical and behavioral knowledge *relevant to health and disease* is the defining characteristic of behavioral medicine. Clearly, this issue needs further deliberation and discussion. In order to give direction to this discussion, we would propose the scope of behavioral medicine to extend ‘from bio-behavioral mechanisms (i.e. the interaction *among* biomedical, psychological, social, societal, cultural and environmental processes *related to health and disease*), to clinical diagnosis and intervention, and to public health’.

Consistent with the definition of behavioral medicine which refers to ‘the development and integration of biomedical and behavioral knowledge’, “behavioral” refers to a wide range of processes (psychological, social, societal, cultural, and environmental processes). The term “psychological processes” refers to emotions and cognitions as well as behaviors in a more narrow sense, such as smoking, physical activity, and diet. In order to avoid using “behavior” with two different meanings (a wide and a narrow meaning), we use “psychological” to refer to emotions, cognitions and behavior-in-a-more-narrow-sense.

Johnston and Johnston [3] argue that since the original development of the definition “there is more ‘behavior’ in behavioral medicine”. They argue that there has been an increasing focus on behavior as a cause and consequence of health status, to complement the earlier emphasis on stress, emotions, beliefs, traits, and mental health. This leads them to emphasize “behavior” in the scope: “…. to *behavioral* processes in clinical diagnosis and intervention, and in public health”. We fully agree that the emphasis on behavior and behavior change techniques has increased, and we agree that this is a very important development. However, we would prefer not to narrow down the scope to overemphasize behavior as biology, emotions, beliefs, traits, and mental health are highly relevant aspects of clinical diagnosis and intervention, and public health.

### Application

In line with the original definition, we proposed the application of knowledge as a defining characteristic of behavioral medicine. We suggested several minor adaptations, leading to the following proposal: ‘… and the application of this knowledge to prevention, health promotion, diagnosis, treatment, rehabilitation, and care’ [1].

We suggested to delete “etiology” from the original list. Both Weiss [2] and Johnston and Johnston [3] object to this proposal, emphasizing the importance of behavioral factors in the etiology of disease. We fully agree on the important role of behavioral factors in the development of disease. Above, we have emphasized that a wide range of behavioral factors play a role in the causation of disease, in the context of the discussion on bio-behavioral mechanisms. However, we suggested to delete “etiology” from the areas where behavioral medicine is being *applied*, as “etiology” is not area where knowledge is being applied: Etiology is not an intervention. Prevention, health promotion, diagnosis, treatment, and rehabilitation all concern interventions. Etiology is in a different category, related to knowledge on causal mechanisms, and in our opinion does not fit here. Nevertheless, we fully agree on the importance of behavioral factors in the etiology of disease.

Weiss [2] proposed not to include public health, as it seems that we would be “overreaching” to include this field as a subset of behavioral medicine. In view of the recent development of the field, we do feel that public health needs to be listed. Attempts to control the obesity epidemic are just one example of how behavioral medicine knowledge is being applied in public health. Similarly, Weiss [2] questions whether “care” should be listed. On the other hand, Kawakami [6] argued in favor of adding care, in the context of improving well-being and quality of life. We suggest that care is indeed an area where behavioral medicine knowledge makes important contributions to well-being and quality of life.

### Towards a Definition of Behavioral Medicine

Our colleagues raised several valuable issues which we agree need further discussion. They also pointed to the need to ask input from the ISBM member societies [2, 4]. We gratefully accept the suggestion to seek the views from our members. We will propose to the ISBM Governing Council to start that process. Based on the comments of our esteemed colleagues and our reflection on those comments, we offer the following refined definition as the starting point for that process:

*‘Behavioral medicine can be defined as the field characterized by the collaboration among multiple disciplines concerned with the development and integration of biomedical and behavioral knowledge relevant to health and disease, and the application of this knowledge to prevention, health promotion, diagnosis, treatment, rehabilitation, and care. The scope of behavioral medicine extends from bio-behavioral mechanisms (i.e. the interaction among biomedical, psychological, social, societal, cultural and environmental processes related to health and disease), to clinical diagnosis and intervention, and to public health’.*

## References

[1] Dekker J, Stauder A, Penedo FJ. Proposal for an update of the definition and scope of behavioral medicine. International Journal of Behavioral Medicine. Doi:10.1007/s12529-016-9610-7 (in this issue). 10.1007/s12529-016-9610-7 PMC5288409 27844398

[2] Weiss SM. Proposal for an update of the definition and scope of behavioral medicine: commentary. International Journal of Behavioral Medicine. Doi:10.1007/s12529-016-9614-3 (in this issue). 10.1007/s12529-016-9614-3 27905007

[3] Johnston M, Johnston D. What is behavioral medicine? Commentary on definition proposed by Dekker, Stauder & Penedo International Journal of Behavioral Medicine. Doi:10.1007/s12529-016-9611-6 (in this issue). 10.1007/s12529-016-9611-6 PMC5288408 27924552

[4] Lau JT. Commentary: proposal for an update of the definition and scope of behavioral medicine. International Journal of Behavioral Medicine. Doi:10.1007/s12529-016-9615-2 (in this issue). 10.1007/s12529-016-9615-2 27981473

[5] Nater UM. Behavioral medicine and related fields. International Journal of Behavioral Medicine. Doi:10.1007/s12529-016-9613-4 (in this issue). 10.1007/s12529-016-9613-4 27844399

[6] Kawakami N. Reflections on the proposed definition and scope of behavioral medicine. International Journal of Behavioral Medicine. Doi:10.1007/s12529-016-9612-5 (in this issue). 10.1007/s12529-016-9612-5 27900732

[7] Choi BC, Pak AW (2006). Multidisciplinarity, interdisciplinarity and transdisciplinarity in health research, services, education and policy: 1. definitions, objectives, and evidence of effectiveness. Clin Invest Med.

